# Role of Salt-Inducible Kinase 1 in the Activation of MEF2-Dependent Transcription by BDNF

**DOI:** 10.1371/journal.pone.0054545

**Published:** 2013-01-22

**Authors:** Charles Finsterwald, Anthony Carrard, Jean-Luc Martin

**Affiliations:** Center for Psychiatric Neuroscience, Department of Psychiatry-CHUV, Prilly-Lausanne, Switzerland; Rutgers University, United States of America

## Abstract

Substantial evidence supports a role for myocyte enhancer factor 2 (MEF2)-mediated transcription in neuronal survival, differentiation and synaptic function. In developing neurons, it has been shown that MEF2-dependent transcription is regulated by neurotrophins. Despite these observations, little is known about the cellular mechanisms by which neurotrophins activate MEF2 transcriptional activity. In this study, we examined the role of salt-inducible kinase 1 (SIK1), a member of the AMP-activated protein kinase (AMPK) family, in the regulation of MEF2-mediated transcription by the neurotrophin brain-derived neurotrophic factor (BDNF). We show that BDNF increases the expression of SIK1 in primary cultures of rat cortical neurons through the extracellular signal-regulated kinase 1/2 (ERK1/2)-signaling pathway. In addition to inducing SIK1 expression, BDNF triggers the phosphorylation of SIK1 at Thr182 and its translocation from the cytoplasm to the nucleus of cortical neurons. The effects of BDNF on the expression, phosphorylation and, translocation of SIK1 are followed by the phosphorylation and nuclear export of histone deacetylase 5 (HDAC5). Blockade of SIK activity with a low concentration of staurosporine abolished BDNF-induced phosphorylation and nuclear export of HDAC5 in cortical neurons. Importantly, stimulation of HDAC5 phosphorylation and nuclear export by BDNF is accompanied by the activation of MEF2-mediated transcription, an effect that is suppressed by staurosporine. Consistent with these data, BDNF induces the expression of the MEF2 target genes Arc and Nur77, in a staurosporine-sensitive manner. In further support of the role of SIK1 in the regulation of MEF2-dependent transcription by BDNF, we found that expression of wild-type SIK1 or S577A SIK1, a mutated form of SIK1 which is retained in the nucleus of transfected cells, is sufficient to enhance MEF2 transcriptional activity in cortical neurons. Together, these data identify a previously unrecognized mechanism by which SIK1 mediates the activation of MEF2-dependent transcription by BDNF.

## Introduction

Salt-inducible kinase 1 (SIK1) is a member of the AMP-activated protein kinase (AMPK) family of Ser/Thr kinases originally identified from the adrenal glands of high salt diet-treated rats [1]. In the central nervous system, SIK1 is expressed in striatal, cortical, hippocampal and hypothalamic neurons [2], [3], [4], [5]. The expression of SIK1 was found to be regulated by neuronal activity *in vitro* and *in vivo* [3], [4], [5]. In addition to SIK1, the SIK family includes two other members, i.e. SIK2 and SIK3. SIK2 is predominantly expressed in adipose tissue and is involved in neuronal survival [6], [7] and in the suppression of melanogenesis in melanocytes [8], [9]. The ubiquitously expressed SIK3 isoform induces chondrocyte differentiation [10] and regulates glucose and lipid metabolism [11].

SIK1 was initially shown to act as a repressor of cAMP-dependent transcription of steroidogenic enzymes [12], and was later found to repress cAMP response element-binding protein (CREB) transcriptional activity by phosphorylating CREB-regulated transcription coactivators (CRTCs) [13], [14]. Thus, phosphorylation of CRTCs by SIK1 triggers nuclear export and cytoplasmic sequestration of CRTCs, thereby preventing activation of CREB-mediated transcription [14], [15].

More recently, SIK1 was shown to promote the phosphorylation of class II histone deacetylases (HDACs) [16], [17] that repress transcription by associating with a variety of transcription factors and corepressors [18]. In particular, SIK1 was found to phosphorylate and inactivate class II HDACs in skeletal muscle, thus enhancing myocyte enhancer factor 2 (MEF2) transcriptional activity and expression of MEF2 target genes [17]. Similarly, KIN-29, the homolog of SIK in *C. elegans*, was shown to phosphorylate and inactivate class II HDACs in chemosensory neurons, thereby upregulating chemoreceptor gene expression via the *C. elegans* MEF2 ortholog MEF-2 [16]. Together, these studies provide evidence that phosphorylation of class II HDACs by SIK1 leads to the activation of MEF2-dependent transcription in different cell types.

There is compelling evidence supporting a role for MEF2 transcription factors in neuronal survival, differentiation and synapse development [19], [20]. For instance, inhibition of MEF2 activity causes apoptotic cell death in cortical neurons, suggesting that MEF2-dependent transcription is necessary for neuronal survival [21]. In addition, activation of MEF2-dependent transcription was found to regulate synapse elimination in the hippocampus and cerebellum [22], [23], [24].

In developing neurons, MEF2-mediated transcription is regulated by a number of stimuli such as calcium influx resulting from neuronal activity [22], [23] and neurotrophin-mediated signaling [22], [23], [25], [26], [27], [28]. In particular, the neurotrophin brain-derived neurotrophic factor (BDNF) was shown to activate MEF2-mediated transcription in cerebellar granule and cortical neurons [25], [26], [27], [29].

Together, these observations led us to examine the role of SIK1 in the regulation of MEF2-dependent transcription by BDNF. In the present study, we show that BDNF induces the expression, phosphorylation and, translocation of SIK1 in cortical neurons. These effects of BDNF are followed by the phosphorylation and nuclear export of HDAC5, leading to the activation of MEF2-mediated transcription.

## Materials and Methods

### Cortical Neuron Culture

Primary cultures of cortical neurons were prepared from 18-day-old Sprague Dawley rat embryos, as previously described [30]. Dissociated cells were plated at a density of 60,000 cells/cm^2^ on poly-D-lysine (Sigma-Aldrich) coated dishes or glass coverslips, and were cultured in Neurobasal medium (Invitrogen) supplemented with B-27 (Invitrogen), 0.5 mM glutamine (Sigma-Aldrich) and 2 µM glutamate (Sigma-Aldrich). Cortical neurons were maintained for 4 days at 37°C in a humidified atmosphere consisting of 95% air and 5% CO_2_.

### Ethics Statement

All experiments complied with the Swiss federal act on animal protection and the Swiss animal protection ordinance, and were approved by the veterinary office of Canton de Vaud (Lausanne, Switzerland, permit Number 684.9).

### Cell Stimulation

After 4 days *in vitro*, cortical neurons were treated with 10 ng/ml BDNF (Alomone Labs). When indicated, neurons were also exposed to 50 µM U0126 (Calbiochem), 10 µM LY294002 (Calbiochem), 10 µM KN-62 (Tocris Bioscience), or 10 nM staurosporine (LC Laboratories) 30 min before stimulation with BDNF. U0126, LY294002, KN-62 and staurosporine were solubilized in dimethyl sulfoxide (DMSO), and the same volume of DMSO (final concentration 0.1%) was added into the medium of non-treated cultures as vehicle.

### Lipofection

For immunocytochemistry experiments, cortical neurons grown on glass coverslips were transfected after 3 days *in vitro* with pCAG-GFP (Addgene plasmid # 11150) [31], pCDNA3.1-HDAC5-FLAG (Addgene plasmid # 13822) [32], pGFPC-rat SIK1 [13], or pGFPC-rat S577A SIK1 [13] using Lipofectamine 2000 reagent (Invitrogen), according to the manufacturer’s instructions. pGFPC-rat SIK1 and pGFPC-rat S577A SIK1 were kindly provided by Dr H. Takemori. For MEF2-luciferase assay experiments, cortical neurons were transfected 5 h after plating using Lipofectamine 2000 reagent with pMEF2FLuc [33], pUB6-lacZ [34], and when indicated with pCAG-GFP, pGFPC-rat SIK1, or pGFPC-rat S577A SIK1.

### Nucleofection

Nucleofection of pGFPC-rat SIK1 [13] was performed on 5×10^6^ dissociated cortical neurons using the rat neuron nucleofector kit and the nucleofector device (Amaxa Biosystems, Lonza, Switzerland), according to the manufacturer’s instructions. Transfected cells were plated at a density of 100,000 cells/cm^2^.

### Real-time Quantitative PCR Analysis

Total RNA was isolated from 4 days *in vitro* cortical neurons using RNeasy Plus Mini kit (Qiagen), according to the manufacturer’s instructions. 200 ng of total RNA was used for reverse transcription into first-strand cDNA with TaqMan reverse transcription reagents (Applied Biosystems). The resulting cDNA was then amplified by real-time quantitative PCR using Power SYBR Green PCR Master Mix (Applied Biosystems) and a 7500 Real-Time PCR System (Applied Biosystems). The following primer pairs were used for specific mRNA amplification: Arc, forward 5′-GAA CGA CAC CAG GTC TCA AG-3′, reverse 5′-TTT CTC TGC CTT GAA AGT GTC-3′; Gapdh, forward 5′-ATG TAT CCG TTG TGG ATC TGA CAT-3′, reverse 5′-ACC TGC TTC ACC ACC TTC TTG A-3′; Nur77, forward 5′-CGG AGA TGC CCT GTA TCC-3′, reverse 5′-ATG GTG GGC TTG CTG AAC-3′; Sik1, forward 5′-AGC TTT ACC AGG TTA TGG AG-3′, reverse 5′-AGT CAG ATA ATC GAA CAT TTC TC-3′. The analysis of relative mRNA levels was performed using a delta-C_T_ (ΔΔC_t_) relative quantification model [35] with gapdh as a reference gene. Each value is expressed as fold change relative to the mRNA level of the control value.

### Western Blot Analysis

Western blot analysis was performed, as previously described [30], with rabbit anti-SIK1 (1∶5000) and rabbit anti-phospho-Thr182 SIK1 (1∶1000) antibodies generously provided by Dr H. Takemori (National Institute of Biomedical Innovation, Osaka, Japan), rabbit anti-phospho-Ser259 HDAC5 antibody (1∶1000; Abcam) and mouse anti-β-tubulin antibody (1∶1000, Sigma-Aldrich). ECL horseradish peroxidase-conjugated anti-rabbit and anti-mouse antibodies (1∶10,000; Amersham Biosciences) were used as secondary antibodies. The total densitometric value of each band was quantified with ImageJ software (National Institute of Health), normalized to the corresponding β-tubulin level, and expressed as fold change relative to the control value.

### Immunocytochemistry

After 4 days *in vitro*, cortical neurons grown on glass coverslips were processed for immunocytochemistry, as previously described [36]. Neurons were immunostained with mouse anti-FLAG M2 (1∶1000, Sigma Aldrich), mouse anti-MAP2 (1∶1000, Sigma Aldrich), and rabbit anti-GFP (1∶500, Invitrogen) antibodies. Alexa Fluor 546 goat anti-mouse and Alexa Fluor 488 goat anti-rabbit (Invitrogen) were used as secondary antibodies. Cells were then counterstained with DAPI (Invitrogen), and glass coverslips were mounted with Prolong Gold antifade reagent (Invitrogen) on microscope slides. Images were analyzed with an Axioplan 2 fluorescence microscope (Zeiss). To characterize the subcellular localizations of HDAC5-FLAG and SIK1-GFP, FLAG and GFP immunofluorescence intensities were quantified with ImageJ software in the cytoplasmic and nuclear compartments of transfected neurons. The cytoplasmic and nuclear compartments of neurons co-transfected with pCAG-GFP and HDAC5-FLAG were delineated using GFP immunostaining and DAPI counterstaining, respectively and with MAP2 immunostaining and DAPI counterstaining for neurons transfected with SIK1-GFP. Data are shown as the ratio of HDAC5-FLAG or SIK1-GFP immunofluorescence intensity in the nucleus to the cytoplasm of transfected neurons.

### MEF2-luciferase Assay

Cortical neurons at 4 days *in vitro* were lysed in luciferase cell culture lysis reagent (Promega) and processed for luciferase activity with the luciferase assay system (Promega) and a Turner-Designs TD-20/20 luminometer (DLReady), according to the manufacturer’s instructions. Lysed cells were also processed for β-galactosidase activity using Galacto-Light chemiluminescent reporter gene assay (Applied Biosystems). Data are expressed as the ratio of luciferase activity to β-galactosidase activity, and are shown as fold changes relative to the control value.

### Statistical Analysis

Data are shown as the mean ± SEM of n values, from at least three independent experiments. Statistical significance was analyzed with one-way ANOVA followed by Bonferonni’s or Dunnett’s post hoc test using PASW-SPSS 18.0 software (IBM). The statistical significance is shown as *p<0.05, **p<0.01, ***p<0.001, and NS p>0.05.

## Results

**Figure 1:**
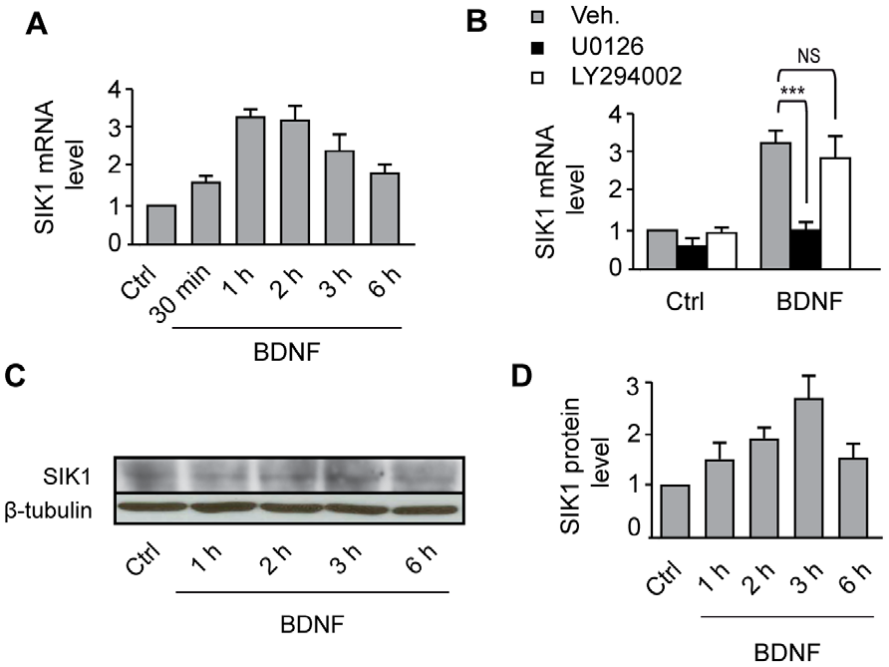
BDNF enhances the expression of SIK1 through the MAPK signaling pathway. **A**, SIK1 mRNA expression was analyzed by real-time quantitative PCR in cortical neurons treated with BDNF for the indicated time points. Results represent the mean ± SEM of 3–6 independent experiments. **B**, The expression of SIK1 mRNA was analyzed by real-time quantitative PCR in neurons treated with BDNF for 1 h in the presence of the MEK1/2 inhibitor U0216, the PI3K inhibitor LY294002, or the vehicle (Veh). Results are the mean ± SEM of 3 independent experiments. ***p<0.001; NS, not significantly different. **C**, Western blot analysis of SIK1 protein expression in neurons treated with BDNF. **D**, SIK1 protein levels are expressed as fold changes relative to the control (Ctrl) value, and are the mean ± SEM of 5–8 independent experiments.

**Figure 2:**
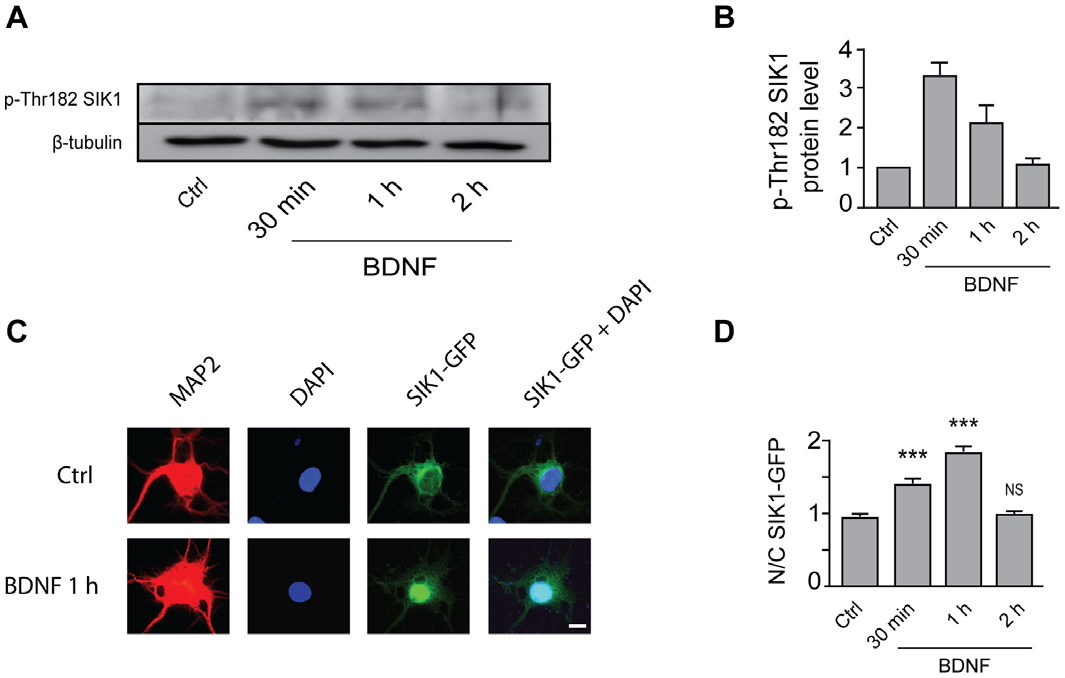
BDNF induces phosphorylation and nuclear translocation of SIK1. **A**, Western blot analysis of phospho-Thr182 SIK1 (p-Thr182 SIK1) expression in cortical neurons transfected with SIK1-GFP and treated with BDNF. **B**, p-Thr182 SIK1 protein levels are expressed as fold changes relative to the control (Ctrl) value, and are the mean ± SEM of 3 independent experiments. **C**, Cortical neurons were transfected with SIK1-GFP and treated with BDNF. After treatment, cortical neurons were immunostained for GFP and MAP2, followed by DAPI counterstaining. Scale bar = 10 µm. **D**, Quantitative analysis of the nuclear/cytoplasmic ratio (N/C) of SIK1-GFP immunofluorescence in Ctrl and BDNF-treated cortical neurons. Cytoplasmic and nuclear compartments were determined with MAP2 immunostaining and DAPI counterstaining, respectively. Results are the mean ± SEM of at least 54 neurons from 4 independent experiments. ***p<0.001 and NS, not significantly different, compared to Ctrl by one-way ANOVA followed by Dunnett’s post hoc test.

As a first step towards assessing the role of SIK1 in the activation of MEF2-dependent transcription by BDNF, we examined the regulation of SIK1 expression by BDNF. We found that treatment of cortical neurons with BDNF increases SIK1 mRNA levels (Fig. 1A) and protein expression (Fig. 1C–D). The increase in SIK1 mRNA levels induced by BDNF was completely abolished by treatment with the MEK1/2 inhibitor U0126, whereas inhibition of the PI3K signaling pathway by LY294002 did not affect BDNF-induced increases in SIK1 mRNA levels (Fig. 1B). These data indicate that BDNF induces SIK1 expression via a MEK1/2-dependent, PI3K-independent mechanism. Because there is evidence that SIK1 is activated through phosphorylation at Thr182 [37], we examined whether BDNF regulates SIK1 Thr182 phosphorylation. Our data revealed that treatment of cortical neurons with BDNF induced a transient phosphorylation of SIK1 at Thr182 (Fig. 2A–B). In addition, phosphorylation of SIK1 by BDNF was accompanied by the translocation of SIK1 from the cytoplasm to the nucleus of cortical neurons (Fig. 2C–D). Together, these data show that BDNF induces the expression, phosphorylation and, translocation of SIK1 in cortical neurons.

**Figure 3:**
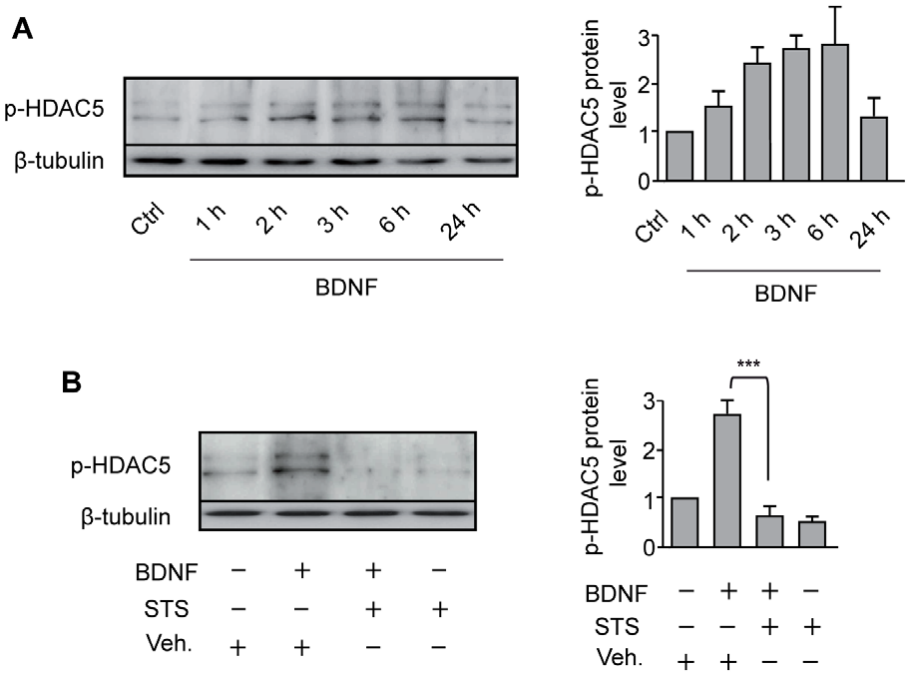
Stimulation of HDAC5 phosphorylation by BDNF is mediated by a staurosporine-sensitive mechanism. **A**, Left panel, western blot analysis of phospho-Ser259 HDAC5 (p-HDAC5) expression in cortical neurons treated with BDNF. Right panel, p-HDAC5 protein levels are presented as fold changes relative to the control (Ctrl) value and are the mean ± SEM of 4–6 independent experiments. **B**, Left panel, western blot analysis of p-HDAC5 expression in neurons treated with BDNF for 3 h in the presence of staurosporine (STS) or vehicle (Veh). Right panel, quantitative analysis of p-HDAC5 protein levels. Data are the mean ± SEM of 4–5 independent experiments. ***p<0.001.

**Figure 4:**
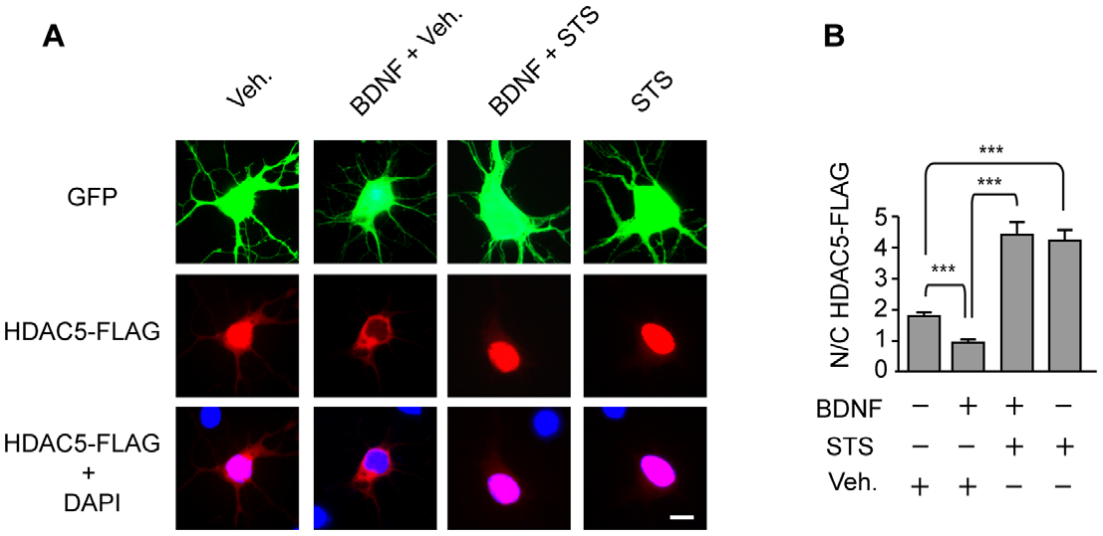
Effects of BDNF and staurosporine on the subcellular localization of HDAC5. **A**, Cortical neurons expressing GFP and HDAC5-FLAG were treated with BDNF for 3 h in the presence of staurosporine (STS) or vehicle (Veh). After treatment, cells were immunostained for GFP and FLAG, followed by DAPI counterstaining. Scale bar = 10 µm. **B,** Quantitative analysis of the nuclear/cytoplasmic ratio (N/C) of HDAC5-FLAG immunofluorescence in cortical neurons treated with BDNF and STS. Nuclear and cytoplasmic compartments were determined with DAPI counterstaining and GFP immunostaining, respectively. Results represent the mean ± SEM of at least 55 neurons from 3 independent experiments. ***p<0.001.

Because there is evidence that SIK1 triggers HDAC5 phosphorylation in skeletal muscle [17], we next examined whether induction of SIK1 expression, phosphorylation and, nuclear translocation by BDNF was accompanied by changes in the phosphorylation of HDAC5 at Ser259. Our data revealed that exposure of cortical neurons to BDNF increases phospho-Ser259 HDAC5 expression (Fig. 3A). This effect was suppressed by addition of a low concentration (10 nM) of staurosporine (Fig. 3B), a Ser/Thr kinase inhibitor that blocks SIK activity [15]. As phosphorylation of HDAC5 is known to trigger its nuclear export, we examined whether HDAC5 phosphorylation induced by BDNF leads to its subcellular relocalization in cortical neurons. Using an HDAC5-FLAG protein to monitor changes in cellular localization, we found that HDAC5-FLAG was targeted predominantly to the nucleus of cortical neurons in naive cultures, whereas treatment with BDNF induced the redistribution of HDAC5-FLAG to the cytoplasm of transfected neurons (Fig. 4A–B). Consistent with the important role of SIK1 in the nuclear-to-cytoplasmic shuttling of class II HDACs [17], blockade of SIK activity by staurosporine resulted in the strong nuclear accumulation of HDAC5-FLAG in cortical neurons exposed or not to BDNF (Fig. 4A–B). These data provide evidence that activation of SIK1 by BDNF induces the phosphorylation and nuclear export of HDAC5 in cortical neurons.

**Figure 5:**
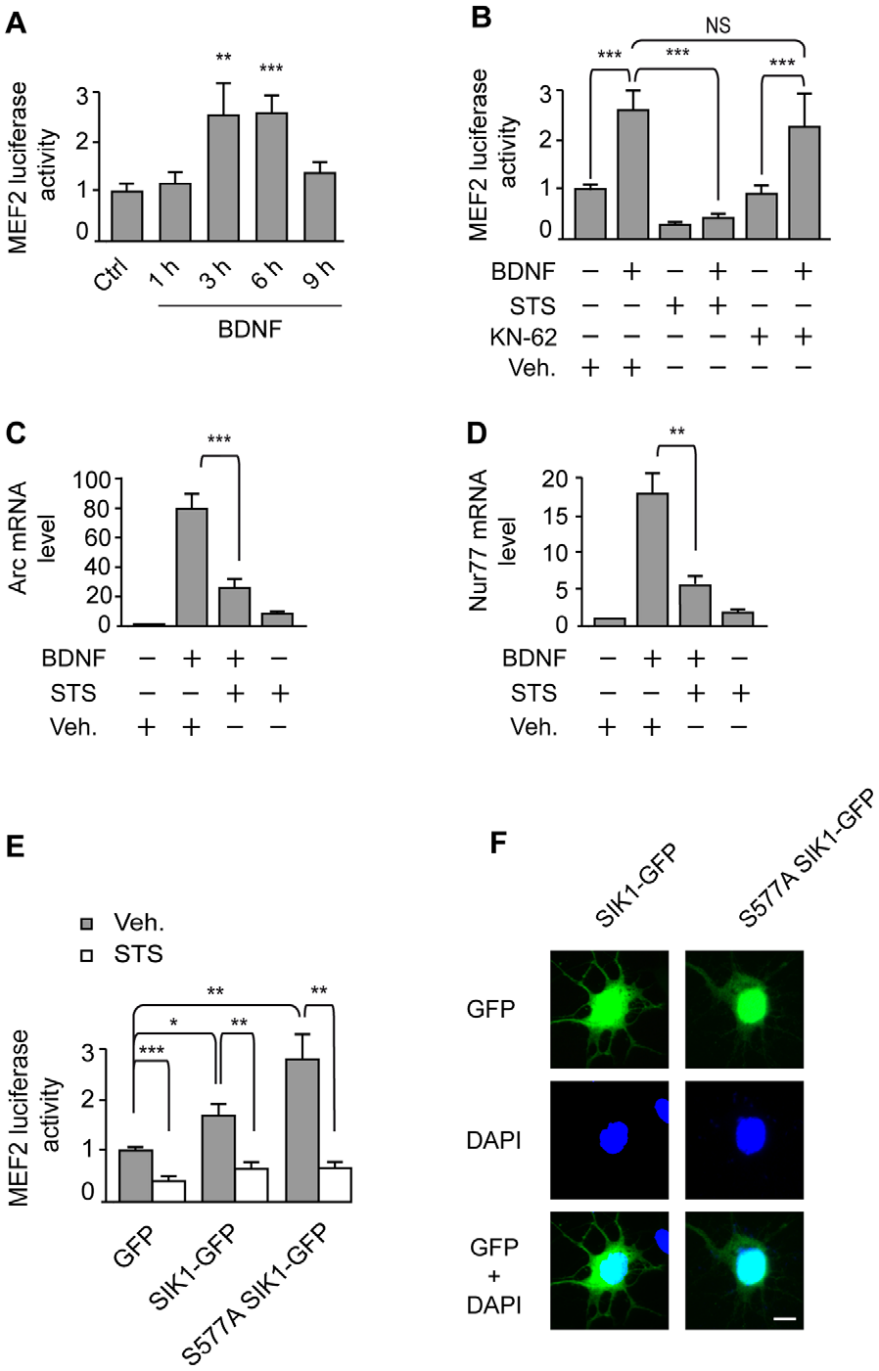
Role of SIK1 in the induction of MEF2-dependent transcription by BDNF. **A**, Cortical neurons transfected with pMEF2FLuc and pUB6-lacZ were treated with BDNF for the indicated time points. Results represent MEF2-luciferase activity normalized to β-galactosidase activity, and are expressed as fold changes relative to the control (Ctrl) value. Data are the mean ± SEM of at least 6 determinations from 3–5 independent experiments. ***p<0.001, **p<0.01 compared with the control (Ctrl) value. **B**, Cortical neurons transfected with pMEF2FLuc and pUB6-lacZ were treated with BDNF for 6 h in the presence of staurosporine (STS), KN-62 or vehicle (Veh). MEF2 luciferase activity is expressed as fold changes relative to MEF2 activity in neurons treated with Veh. Data are the mean ± SEM of 7–10 determinations from 3–5 independent experiments. **C–D**, The expression of Arc (C) and Nur77 (D) mRNAs was analyzed by real-time quantitative PCR after treatment of cortical neurons with BDNF for 1 h in the presence of STS or Veh. Results are the mean ± SEM of 4 independent experiments. **E**, Cortical neurons were transfected with pMEF2FLuc and pUB6-lacZ along with plasmids encoding GFP, SIK1-GFP, or S577A SIK1-GFP, and were then exposed to STS or Veh. for 6 h. Data are expressed as in (A) and (B), and are the mean ± SEM of 10–13 determinations from 4 independent experiments. ***p<0.001, **p<0.01, *p<0.05. **F**, Cortical neurons transfected with SIK1-GFP and S577A SIK1-GFP were immunostained for GFP followed by DAPI counterstaining. Scale bar = 10 µm.

Class II HDACs interact directly with MEF2 transcription factors to repress gene expression in skeletal muscle [38]. However, phosphorylation and subsequent nuclear export of class II HDACs leads to the derepression of MEF2-dependent transcription [39]. These observations led us to investigate whether the effect of BDNF on HDAC5 phosphorylation and nuclear export was accompanied by activation of MEF2-mediated transcription. Using a MEF2-luciferase reporter assay to monitor MEF2 activity, we found that treatment of cortical neurons with BDNF significantly increased MEF2 transcriptional activity (Fig. 5A), whereas BDNF-induced MEF2 transcriptional activity was completely suppressed by staurosporine (Fig. 5B). Because it has been shown that activation of MEF2 is also mediated by CaMKII [40], [41], we examined whether inhibition of CaMKII by KN-62 alters the activation of MEF2 by BDNF. We found that KN-62 did not affect BDNF-induced MEF2 activity (Fig. 5B), ruling out the involvement of CaMKII in the activation of MEF2-dependent transcription by BDNF. These data provide evidence that the induction of SIK1 expression, phosphorylation and, nuclear translocation by BDNF and the consequent stimulation of HDAC5 phosphorylation and nuclear export leads to the induction of MEF2 transcriptional activity.

To further characterize the role of SIK1 in the regulation of MEF2-mediated transcription by BDNF, we examined the regulation of the expression of the prototypical MEF2 target genes Arc and Nur77 [22], [42] by BDNF and staurosporine. Consistent with the regulation of MEF2 activity, our data revealed that BDNF induces the upregulation of Arc and Nur77, an effect abolished after inhibition of SIK1 activity by staurosporine (Fig. 5C–D).

To further support the role of SIK1 in the regulation of MEF2-dependent transcription by BDNF, we examined MEF2 activity in transfected neurons expressing wild-type SIK1 or a mutated form of SIK1, S577A SIK1, which is retained in the nucleus of transfected cells [13]. These data revealed that expression of wild-type SIK1 and S577A SIK1 are sufficient to stimulate MEF2 transcriptional activity (Fig. 5E). However, treatment of cortical neurons with staurosporine abolished the effects of expressing wild-type SIK1 and S577A SIK1 on MEF2 activity (Fig. 5E). Interestingly, expression of S577A SIK1 has a greater effect on MEF2 transcriptional activity than expression of wild-type SIK1 (Fig. 5E), which is consistent with the increased nuclear localization of the mutant compared to the wild-type form of SIK1 (Fig. 5F). Together, these data unveil the critical role of SIK1 in the regulation of MEF2 transcriptional activity by BDNF.

## Discussion

Data from this study show that BDNF stimulates MEF2 transcriptional activity through a novel molecular mechanism. Thus, by inducing the expression, phosphorylation, and nuclear translocation of SIK1, BDNF phosphorylates HDAC5 and triggers its nuclear export resulting in enhanced MEF2-dependent transcription in cortical neurons.

Previous studies in different cell types have shown that the expression of SIK1 is upregulated by the cAMP/PKA signaling pathway. For instance, forskolin induces SIK1 expression in primary myocytes, C2C12 myoblasts and hypothalamic neurons [4], [17]. Other studies have reported that the β-adrenergic receptor agonist isoproterenol upregulates SIK1 expression in skeletal muscle and pinealocytes [17], [43]. In developing cortical neurons, evidence indicates that membrane depolarization by KCl induces SIK1 expression, an effect that is abolished by the L-type voltage-gated calcium channel blocker nifedipine [3]. In the present study we show, for the first time, that the expression of SIK1 is upregulated in developing neurons by the neurotrophic factor BDNF (Fig. 1).

In addition to inducing SIK1 expression, BDNF triggers the phosphorylation and nuclear translocation of SIK1 (Fig. 2). Interestingly, it has been shown that phosphorylation of SIK1 at Thr182 is mediated by the liver kinase B1 (LKB1) [37], [44], suggesting that BDNF increases SIK1 phosphorylation at Thr182 by activating LKB1. Consistent with this hypothesis, BDNF has been shown to stimulate LKB1 activity in cortical and hippocampal neurons [45], [46]. In addition, BDNF-induced SIK1 phosphorylation at Thr182 is associated with its nuclear translocation (Fig. 2C–D). In line with these findings, it is interesting to note that intraperitoneal injection of cocaine induces both phosphorylation of SIK1 at Thr182 and translocation of SIK1 from the cytoplasm to the nucleus of rat striatal neurons [2].

Importantly, our data show that induction of SIK1 expression, phosphorylation and, nuclear translocation by BDNF is followed by the increased phosphorylation and nuclear export of HDAC5 (Fig. 3–4), as well as by the activation of MEF2 transcriptional activity (Fig. 5A) and MEF2-dependent gene expression (Fig. 5C–D). Interestingly, previous studies have shown that the SIK1-HDAC5-MEF2 pathway is also activated in skeletal and cardiac muscle cells. Thus, in skeletal myocytes and C2C12 myoblasts, induction of SIK1 expression by forskolin or isoproterenol increases the phosphorylation and nuclear export of HDAC5, thereby enhancing MEF2-dependent transcription [17]. In line with these data, overexpression of SIK1 was found to induce nuclear export of HDAC5 and activation of MEF2C in C2C12 myoblasts [47]. In addition, recent evidence indicates that induction of SIK1 expression and activity by increases in intracellular sodium concentration in a cardiac myocyte cell line activates MEF2 through HDAC5 phosphorylation [48]. Together, these observations provide evidence that regulation of MEF2 activity by the induction of SIK1 expression and phosphorylation of HDAC5 is not limited to neuronal cells but extend to other cell types such as skeletal and cardiac muscle cells.

The transcriptional activity of MEF2 is regulated by direct phosphorylation and by indirect mechanisms [19], [39], [49]. In particular, activation of p38 MAPK and ERK5 was shown to increase MEF2 transcriptional activity through the phosphorylation of MEF2 transactivation domain [19], [39], [49]. In addition, it is well established that phosphorylation of class II HDACs leads to their nuclear-to-cytoplasmic shuttling and to the subsequent derepression of MEF2 [39], [49]. Although most of these studies are related to skeletal muscle differentiation or cardiac growth [18], there is also evidence that KCl-induced depolarization of cerebellar granule and hippocampal neurons induces nuclear export of HDAC5, resulting in increased MEF2 activity [40], [50]. In this context, our study provides the first evidence of the stimulation of MEF2 activity by a neurotrophic factor through the increased phosphorylation and nuclear export of HDAC5 (Fig. 3–4, 5A).

Interestingly, BDNF was previously shown to stimulate MEF2-dependent transcription through the activation of ERK5 in cortical and cerebellar granule neurons [25], [26], [27]. More recently, BDNF was found to activate MEF2C-mediated transcription in cortical neurons through ERK1/2-p90 ribosomal S6 kinase 2 (RSK2) signaling pathway [29]. These findings, together with our data, indicate that activation of ERK1/2 by BDNF can stimulate MEF2-dependent transcription by inducing SIK1 expression (Fig. 1) followed by the nuclear export of HDAC5 (Fig. 4) and by the direct phosphorylation of MEF2C by RSK2 [29]. These results support the view that SIK1-mediated inactivation of HDAC5 may act in cooperation with RSK2-dependent phosphorylation of MEF2 to activate MEF2 transcriptional activity in response to BDNF.

In addition to regulating MEF2-dependent transcription, previous studies have revealed an important role for SIK1 in controlling CREB-mediated transcription. Thus, SIK1 inhibits CREB transcriptional activity by phosphorylating CRTCs, triggering their nuclear export and cytoplasmic sequestration [13], [15], [51]. These data provide evidence that MEF2- and CREB-mediated transcription are regulated in opposite directions by SIK1 through the phosphorylation of HDAC5 and CRTCs, respectively.

Together, our data identify a novel signaling pathway by which BDNF stimulates MEF2 activity. Thus, by inducing the expression, phosphorylation and, nuclear translocation of SIK1, BDNF increases HDAC5 phosphorylation and nuclear export, resulting in derepression of MEF2 activity. Because BDNF controls many aspects of neuronal development and synaptic plasticity, characterization of the role of SIK1-mediated activation of MEF2 in the effects of BDNF on neuronal survival, differentiation and synaptic transmission should improve our understanding of the mechanisms by which BDNF regulates neuronal development and function.
